# Therapeutics and Materia Medica

**Published:** 1860-06

**Authors:** 


### REVIEWS AND BIBLIOGRAPHY.

*Therapeutics and Materia Medica: A Systematic Treatise on the Action and Uses of Medicinal Agents, including their Description and History.* By Alfred Stille, M.D., Late Professor of the Theory and Practice of Medicine in the Medical Department of Pennsylvania College, etc., etc. Philadelphia: Blanchard & Lea. 1860. Two Vols., 8vo.

Is a new work on Therapeutics needed? This question the author of the treatise, the title page of which prefixes this article, of course propounded to himself before engaging in the labor of writing two large volumes on the subject. The work itself is his answer to the question. We are of opinion that he was right in answering it affirmatively. Without disparagement of the works which have appeared on this subject within the past few years, it has received far less attention than other departments of medical knowledge, and it claims from medical writers more attention than it has received. We can readily understand that, in surveying the domain of medicine in order to select a portion in which bibliographical labor could be rendered most useful, Dr. Stillé was led to choose the subject of therapeutics. The next question which an author would be likely to ask of himself is, Am I the proper person to write a work on the subject proposed? This question, also, our author virtually answers when he enters on the task of writing the work. Here, too, we believe the author, in this instance, has not misjudged. In fact, among those in our country distinguished as medical writers and teachers, we know of no one more competent to prepare a work on therapeutics, having the character and scope of the one before us, than Dr. Stillé. A man of scholarly attainments, familiar with medical literature, a clear thinker, an elegant writer, a good observer, a conscientious truth-seeker, he possesses the qualities of mind admirably suited to an analytical investigation of our present knowledge of the subject, together with the rare ability of communicating to the reader didactic information in a systematic, lucid, and attractive manner. An examination of the treatise will, if we are not mistaken, afford confirmation of the fitness of the author to the work which he has performed.

We do not intend to write a critical review of Dr. Stillé's treatise. It covers so much ground that the reviewer would require more space than is consistent with the limits of this journal. Nor do we profess to speak of the merits of the treatise after a thorough, careful perusal. Our object in this notice is simply to furnish our readers with an account of its general plan, and the arrangement of topics, with the impression which we have formed after an examination, as yet, of necessity partial.

It is not a treatise on materia medica and therapeutics, but essentially a work on the latter subject; the consideration of medicines, in their physical, chemical, and physiological relations, holding a subordinate place, and, in fact, receiving comparatively little attention. To quote the author's language, the point of view from which the treatise has been written is rather of the bedside of the sick than in the laboratory or the lecture-room. The therapeutical considerations, however, are, for the most part, presented in connection with the different classes of remedies. The Introduction occupies somewhat less than one hundred pages. We should have been glad to have seen a much larger space devoted to General Therapeutics, but we are well aware that this was difficult in a work comprehending the whole range of special therapeutics. The following topics are considered in the introduction: Sources from which medicines are derived; sources of knowledge in therapeutics; sources of knowledge respecting the action of medicines; the physiological action of medicines; the local action of medicines; the remote action of medicines; absorption; the avenues by which medicines are introduced, and the effects which they produce; the curative action of medicines; influences modifying the effects of medicines; the administration of medicines; the art of presenting, and the classification of medicines. These topics are quite sufficient in themselves for a large volume, and, for one, we should be glad to read such a volume from the pen of Dr. Stillé.

Of the foregoing list of topics, that which possesses especial interest and importance is the sources of knowledge in therapeutics. While we accord fully with the views presented by the author under this head, we cannot but regret that he did not consider the topic at greater length. There can hardly be a point in medical philosophy more interesting or important than an inquiry into the modes by which therapeutical knowledge is obtained. We agree with Dr. Stillé when he says, "Experience is really, as well as rationally, the only ground upon which curative effects can be expected from medicines." But in order for this truth to be received by all minds, it would have been well to have entered into a fuller consideration of the grounds on which it rests, and to have shown how it does not conflict with the practical application of physiology and pathology to the study of therapeutics. The plainest and simplest rational inferences from pathological conditions, as to the indications for certain remedies, demand the results of experience to convert these indications into therapeutical principles. Thus, what can apparently be clearer than that an offending substance in the stomach is to be evacuated by an emetic? Yet the fact that certain substances act as emetics, the efficacy of an emetic under these circumstances, the particular kind of emetic to be employed, the mode of administration, &c., are only to be determined by experimental observation. It is equally to be inferred from the knowledge that certain diseases depend on a *materies morbi* in the blood, that it is desirable either to neutralize or eliminate this morbid material; but whether it can be neutralized or eliminated, and if so, by what measures, must be determined by experience, and by experience only. How is medical experience to be obtained? What are the different methods of experimental observation? What the difficulties and liabilities to error in prosecuting these methods? How are the results of experience to be made applicable to individual cases of disease? These and other questions lead to considerations bearing directly, and, if we may use so strong a term in this connection, momentously on medical practice. We know of no one better qualified to discuss these points than Dr. Stillé, and we should have been glad to have seen a much larger space devoted to them.

The author arranges medicines into twelve classes, viz.: Lenitives, astringents, irritants, tonics, general stimulants, cerebro-spinal stimulants, spinants or tetanics, general sedatives, arterial sedatives, nervous sedatives, evacuants, and alteratives. This arrangement is perhaps as good as any that can be framed, with our existing knowledge of the effects and *modus operandi* of different remedies. The several classes are treated of successively, in the order in which they have just been enumerated. First, are considered the general principles of therapeutics involved in each class, and then the results of experience, their action in health and disease, etc., of particular remedies belonging to the class, together with the practical rules regulating their employment in the treatment of diseases. The aim of the author is to present a fair representation of therapeutics in so far as the labors of medical observers of the past and present time afford a basis of facts and principles. As the author justly remarks, "it is impossible in such a work to present in detail the observations, or even the conclusions, of medical writers; and therefore," he adds, "while it has been attempted to furnish a truthful, although a succinct account of both, numerous references to original sources of information are provided for those who desire to prosecute the investigation. These consist of ancient classical authorities, and modern writers of the highest reputation in the Italian, French, German, and English languages, whose observations have been preserved in special essays, or in that great repository of facts, the periodical medical literature of the present century." Mere references constitute a very useful feature of the work. The author says further, (quoting from the preface,) "It seemed as if a treatise executed in so catholic a spirit, and with no conscious bias towards any sect or school, ought to contain a large proportion of solid and useful truth." We believe that it does contain a large proportion of solid and useful truth, and with this belief, we most cordially commend the work to those who may honor this brief notice with a perusal.

